# Levodopa in *Mucuna pruriens* and its degradation

**DOI:** 10.1038/srep11078

**Published:** 2015-06-09

**Authors:** Haridas Pulikkalpura, Rajani Kurup, Paravanparampil Jacob Mathew, Sabulal Baby

**Affiliations:** 1 Plant Genetic Resource Division, Jawaharlal Nehru Tropical Botanic Garden and Research Institute, Pacha-Palode, Thiruvananthapuram 695 562, Kerala, India; 2 Phytochemistry and Phytopharmacology Division, Jawaharlal Nehru Tropical Botanic Garden and Research Institute, Pacha-Palode, Thiruvananthapuram 695 562, Kerala, India

## Abstract

*Mucuna pruriens* is the best known natural source of L-dopa, the gold standard for treatment of Parkinsonism. *M. pruriens* varieties are protein rich supplements, and are used as food and fodder worldwide. Here, we report L-dopa contents in seeds of fifty six accessions of four *M. pruriens* varieties, *M. pruriens* var. *pruriens*, *M. pruriens* var. *hirsuta*, *M. pruriens* var.* utilis* and *M. pruriens* var. *thekkadiensis*, quantified by HPTLC-densitometry. L-dopa contents varied between 0.58 to 6.42 (%, dr. wt.). High and low L-dopa yielding genotypes/chemotypes of *M. pruriens* could be multiplied for medicinal and nutritional purposes, respectively. HPTLC profiles of *M. pruriens* seeds on repeated extraction (24 h) in 1:1 formic acid-alcohol followed by development in butanol:acetic acid:water (4:1:1, v/v) showed consistent degradation of L-dopa (R*f* 0.34 ± 0.02) into a second peak (R*f* 0.41 ± 0.02). An average of 52.11% degradation of L-dopa was found in seeds of *M. pruriens* varieties. Since *M. pruriens* seeds and/or L-dopa are used for treatment of Parkinson’s disease and as an aphrodisiac both in modern and/or traditional systems of medicine, the finding of high level of L-dopa degradation (in pure form and in *M. pruriens* extracts) into damaging quinones and ROS is very significant.

*Mucuna pruriens* (L.) DC. is a climbing legume distributed across the tropics. Four varieties of the species have been documented so far from south India, of which *M. pruriens* var. *pruriens* is well distributed^1^,^2^. *M. pruriens* var. *hirsuta* and *M. pruriens* var. *thekkadiensis* are restricted to the southern parts of the Indian peninsula, and *M. pruriens* var. *utilis* occurs only in cultivation^2^. *M. pruriens* var. *pruriens* and *Mucuna pruriens* var. *utilis* find importance as food, feed, cover crop and fodder and are extensively cultivated worldwide^3^,^4^,^5^,^6^. *M. pruriens* varieties propagate mostly through their seeds. *M. pruriens* var. *pruriens* is best known as the natural source of the aromatic amino acid, L-3,4-dihydroxy phenylalanine (levodopa or L-dopa) (Fig. 1). L-dopa is widely used for the clinical treatment of the neurodegenerative disorder, Parkinson’s disease (PD)^7^,^8^,^9^,^10^,^11^. Seeds of *M. pruriens* var. *pruriens* have long been used in Indian traditional medicine for treatment of PD and also for its aphrodisiac property. *M. pruriens* var. *pruriens* is the only variety of *M. pruriens* extensively studied for its chemical and biological properties^12^,^13^,^14^,^15^,^16^,^17^. L-dopa, 5-methoxy-N,N,dimethyl tryptamine and 5-hydroxy tryptamine (serotonin) are the major therapeutic constituents in *M. pruriens* var. *pruriens*.

PD is characterized by degeneration of dopaminergic neurons in the substantia nigra, and subsequent deficiency of the neurotransmitter dopamine in the brain areas. PD affects motor activities including writing and speaking abilities. Recent studies suggested oxidative stress, mitochondrial dysfunction and impairment of the ubiquitin-proteasome system as the major factors involved in pathogenesis of PD^18^. Patients with PD are treated with L-dopa to improve their motor functions. Dopamine as such does not cross the blood brain barrier whereas L-dopa does, and in the central nervous system dopa decarboxylase converts it into dopamine. Thus L-dopa acts as a precursor to dopamine. So far L-dopa is considered as the gold standard for the treatment of PD and dopamine-responsive dystonia^7^,^8^,^9^,^10^,^11^,^19^. Oxidative stress, caused by oxidation of L-dopa and dopamine, generating semiquinones, quinones, oxygen radicals and other reactive oxygen species (ROS), play a role in neuronal cell death in PD^20^,^21^. Moreover, *O*-quinone products of L-dopa autoxidation are cytotoxic to cellular systems^22^,^23^,^24^,^25^. L-dopa is also an antinutritional factor and its consumption causes vomiting, nausea, abdominal distention, dyskinesia etc^26^. This is due to the conversion of L-dopa into dopamine in the peripheral nervous system by dopa decarboxylase. Moreover, *M. pruriens* var. *pruriens* and L-dopa could recover spermatogenic loss which makes them the treatment of choice for infertility^27^,^28^.

Vachhani *et al*., 2011 and Sundaram and Gurumoorthi, 2012 standardized protocols for HPTLC-based quantification of L-dopa in *M. pruriens* var. *pruriens* seeds^29^,^30^. Modi *et al*., 2008 (5.60%, dr. wt.), Behara *et al*., 2010 (4.83%), Raina and Khatri, 2011 (2.23–5.36%) and Raina *et al*., 2012 (3.29–5.44%) quantified L-dopa contents in *M. pruriens* var. *pruriens* seeds by HPTLC^31^,^32^,^33^,^34^. Mennickent *et al*., 2007^35^ and Vachhani *et al*., 2011^29^ standardized HPTLC-based estimation of L-dopa in pharmaceutical formulations. Kshirsagar *et al*., 2008^36^ (2.11–2.19%), Modi *et al*., 2008^31^ (3.80–4.30%) and Behara *et al*., 2010^32^ (7.48–8.44%) quantified L-dopa contents in marketed formulations and capsules by HPTLC. Shah and Joshi, 2010 estimated L-dopa in *M. pruriens* var. *pruriens* seeds (7.20%) and its formulations (4.20–5.60%) by spectroflourimetry^37^. L-dopa contents in seeds of *Stizolobium pruriens* var. *utilis* (*M. pruriens* var. *utilis*) (3.9–10.6%)^38^, *M. pruriens* var. *utilis* (4.39–5.21%)^5^ and *M. pruriens* var. *pruriens* (4.0–6.0%)^6^ were estimated by HPLC. Soumyanath *et al*., 2012 quantified L-dopa in *M. pruriens* var. *pruriens* formulations (3.0–6.0%) by HPLC^39^. Singh *et al*., 2010 developed HPLC-based quantification of L-dopa in *M. pruriens* var. *utilis*^40^. Jiang *et al*., 2010 carried out HPLC-MS/MS quantification of L-dopa in rat plasma^41^. Dethy *et al*., 1997 determined threshold L-dopa levels in plasma of patients with advanced PD by *in vitro* microdialysis-HPLC^42^.

Most L-dopa quantification studies in *M. pruriens* seeds and formulations by chromatographic techniques (HPTLC, HPLC) are on limited number of samples and involved prolonged, multistep extraction procedures in acidic media. Screening of more *M. pruriens* varieties/accessions could lead to the discovery of high (elite) and low L-dopa yielding accessions suitable for medicinal and nutritional purposes, respectively. Secondly, in our preliminary HPTLC profiling of *M. pruriens* extracts and L-dopa standard, we repeatedly detected labile L-dopa-based degradation signals. Most similar HPTLC/HPLC studies never recorded this degradation signal of L-dopa^5^,^6^,^29^,^31^,^32^,^33^,^34^,^35^,^36^,^38^,^39^,^40^,^41^,^42^. Here we report (i) L-dopa contents in seeds of thirty accessions of the four *M. pruriens* varieties viz., *M. pruriens* var. *pruriens* (21), *M. pruriens* var. *hirsuta* (3), *M. pruriens* var. *utilis* (5) and *M. pruriens* var. *thekkadiensis* (1), collected from various locations in Kerala in south India, (ii) L-dopa contents in second generation seeds of twenty-six accessions of *M. pruriens* var. *pruriens* (21) and *M. pruriens* var. *utilis* (5) grown in an Experimental Plot (EP) under identical ecological conditions, (iii) degradation patterns of L-dopa in *M. pruriens* seed extracts, (iv) quantification of L-dopa degraded products in seeds (30 wild, 26 EP grown accessions) of four *M. pruriens* varieties and (v) characterization of L-dopa degraded moieties by HPTLC, DART-MS and LC/EI-MS.

## Results

### L-dopa contents in *M. pruriens* varieties

L-dopa contents in wild *M. pruriens* var. *pruriens* accessions varied from 0.89 to 6.42 (%, dr. wt.) (Table 1). Percentage contents of second degradation peak (SDP) ranged from 0.1 to 3.85% (%, dr. wt., based on L-dopa calibration) (Table 1). In second generation *M. pruriens* var. *pruriens* seeds grown in the EP, L-dopa contents varied from 0.58 to 4.32%, and % SDP varied from zero (non-detectable) to 3.34%. L-dopa contents in wild accessions of *M. pruriens* var. *hirsuta* ranged from 1.01 to 4.27%, and % SDP ranged from 0.01 to 1.40%. L-dopa contents in seeds of *M. pruriens* var. *utilis* wild accessions varied from 0.65 to 2.67%, and SDP contents varied from 0.12 to 3.82%. In EP grown accessions of *M. pruriens* var. *utilis*, L-dopa contents ranged from 1.33 to 3.97%, and SDP contents were zero to 0.77%. *M. pruriens* var. *thekkadiensis* wild accession showed 4.34% of L-dopa with SDP 0.01% (Table 1). Fruiting was not observed in *M. pruriens* var. *hirsuta* and *M. pruriens* var. *thekkadiensis* in the Field Gene Bank (FGB). This is the first report of L-dopa quantification in *M. pruriens* var. *hirsuta* and *M. pruriens* var. *thekkadiensis*. This is also the first quantitative determination of degradation products of L-dopa in *M. pruriens* seeds.

In wild *M. pruriens* var. *pruriens*, *M. pruriens* var. *hirsuta* and *M. pruriens* var. *utilis* seeds, L-dopa contents did not show any positive correlation with altitudes of their collection locations (Acc. No. 3912, 1 m, L-dopa/SDP 1.63%/1.15%; Acc. No. 4503, 10 m, L-dopa/SDP 1.78%/3.85%; Acc. No. 4091, 802 m, L-dopa/SDP 1.66%/2.96%) (Table 1). *M. pruriens* var. *pruriens* and *M. pruriens* var. *utilis* seeds grown in EP under identical conditions did not show any correlation in L-dopa contents with their parent (wild) accessions (Table 1). Highest L-dopa contents were detected in seeds of wild accessions of *M. pruriens* var. *pruriens* (Acc. No. 4283, 6.42%; Acc. No. 4498, 6.11%), *M. pruriens* var. *thekkadiensis* (Acc. No. 4147, 4.34%) and *M. pruriens* var. *hirsuta* (Acc. No. 3820, 4.27%). Lowest L-dopa percentages were found in EP grown *M. pruriens* var. *pruriens* seeds (Acc. No. 4072, 0.58%; Acc. No. 3822, 0.84%) (Table 1). Again, highest L-dopa yielding *M. pruriens* seeds (Acc. No. 4283, 6.42%; Acc. No. 4498, 6.11%; Acc. No. 4147, 4.34%; Acc. No. 3820, 4.27%) showed lowest degradation levels (Acc. No. 4283, 0.00%; Acc. No. 4498, 0.10%; Acc. No. 4147, 0.01%; 3820, 0.01%). Similarly, lowest L-dopa contents (Acc. No. 4072, 0.58%; Acc. No. 3822, 0.84%; Acc. No. 4100, 0.95%; Acc. No. 4090, 0.97%) were seen in seeds with high levels of degradation (Acc. No. 4072, 1.77%; Acc. No. 3822, 2.93%; Acc. No. 4100, 3.19%; Acc. No. 4090, 2.75%) (Table 1).

Total SDP (72.15%) in wild (30) and EP grown (26) *M. pruriens* accessions was 52.11% of total L-dopa content (138.45%) (Table 1). These data showed that the degradation levels of L-dopa in *M. pruriens* seeds in extraction media (1:1 formic acid-alcohol, 24 h) are highly significant. Standard L-dopa also showed similar degradation into a second signal on HPTLC profile (L-dopa R*f* 0.34** ± **0.02; SDP R*f* 0.41** ± **0.02). Previous studies rarely mentioned^30^ the detection of L-dopa degradation signals on chromatographic profiles of *M. pruriens* seeds and formulations^5^,^6^,^29^,^31^,^32^,^33^,^34^,^35^,^36^,^38^,^39^,^40^,^41^,^42^.

### L-dopa degradation

**Figure 2:**
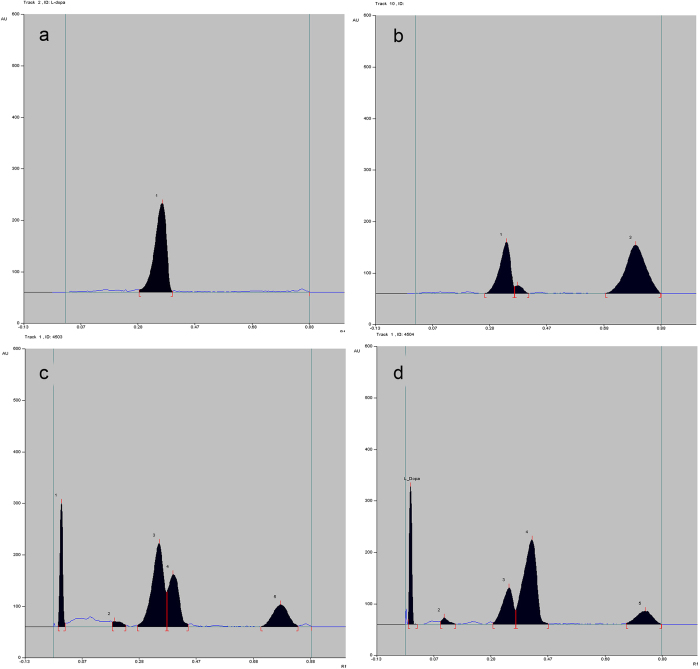
HPTLC profiles of (**a**) L-dopa standard (in 1:1 formic acid-alcohol, fresh), (**b**) degraded L-dopa standard (1:1 formic acid-alcohol, 24 h), (**c**) seed extract of *Mucuna pruriens* var. *pruriens*, accession number 4503 (five step extraction, 1:1 formic acid-alcohol, 24 h) and (**d**) seed extract of *M. pruriens* var. *utilis*, accession number 4504 (five step extraction, 1:1 formic acid-alcohol, 24 h); X-axes - R*f*, Y-axes - AU.

Freshly dissolved L-dopa standard (in 1:1 formic acid-alcohol) on HPTLC showed only one signal at R*f* 0.34** ± **0.02 (Fig. 2a). But, L-dopa standard (decomposed) showed two signals, one at R*f* 0.34** ± **0.02 (L-dopa) and a SDP at R*f* 0.41** ± **0.02 (Fig. 2b). *M. pruriens* seed extracts (wild, EP grown) showed two significant signals (R*f* 0.30–0.40, 0.40–0.46) at varying ratios (Fig. 2c,d). Fresh (24 h extracted in 1:1 formic acid-alcohol) *M. pruriens* var. *pruriens* seed extract, decomposed (24 h extracted, dissolved in 1:1 formic acid-alcohol for seven days) *M. pruriens* var. *pruriens* seed extract and decomposed L-dopa standard showed degradation patterns on HPTLC, DART-MS and LC/EI-MS (Fig. 2, Fig. S1-S7). On DART-MS, fresh L-dopa showed only its own major M+H^+^ signal at 198.09 (Fig. S1). Fresh *M. pruriens* var. *pruriens* extract (24 h extracted) showed M+H^+^ signals at 123.06 (medium), 154.10 (medium, dopamine), 162.07 (medium), 198.09 (only major, L-dopa,), 199.09 (minor) and 224.11 (minor) (Fig. S2). Decomposed *M. pruriens* var. *pruriens* extract (24 h extracted, dissolved in 1:1 formic acid-alcohol for seven days) showed M+H^+^ signals at 129.08 (medium), 176.08 (major), 190.10 (major), 191.11 (minor), 192.13 (minor), 193.13 (minor, dopachrome or 5,6-dihydroxyindole-2-carboxylic acid), 195.17 (minor, dopaquinone, leucodopachrome), 204.11 (major), 218.13 (medium), 244.12 (medium) and 245.13 (minor) (Fig. S3).

Briefly, fresh *M. pruriens* var. *pruriens* extract (24 h extracted) showed only major signals of L-dopa and dopamine. Decomposed *M. pruriens* var. *pruriens* extract did not show L-dopa and dopamine, instead showed a group of degradation signals at the M+H^+^ 190.10, 191.11, 192.13, 193.13 and 195.17. These DART-MS signals correspond to dopachrome, leucodopachrome, dopaquinone, other quinones and ROS (Fig. S3). Decomposed standard L-dopa and decomposed (24 h extracted, dissolved in 1:1 formic acid-alcohol for seven days) *M. pruriens* var. *pruriens* seed extract also showed degradation patterns in LC/EI-MS (Fig. S4-S7).

The degradation patterns of L-dopa in *M. pruriens* var. *pruriens* seed extracts and standard L-dopa in solvent media of 1:1 formic acid-alcohol (acidic), 20 mM Tris buffer (pH 7.2, neutral) and water at various time periods (1 h after initiation of extraction, 1, 7, 30 days after initiation of extraction) were tested through HPTLC profiling. Time dependent degradation was observed in *M. pruriens* var. *pruriens* extracts and in standard L-dopa at these pH values (Fig. S8-S19). The degradation rates of L-dopa in *M. pruriens* var. *pruriens* extracts and standard L-dopa were relatively low at 4 °C.

## Discussion

Our study led to the discovery of elite genotypes/chemotypes in 56 accessions of four *M. pruriens* varieties in wild and EP conditions. L-dopa contents in seeds of *M. pruriens* var. *pruriens* (42), *M. pruriens* var. *utilis* (10), *M. pruriens* var. *hirsuta* (3) and *M. pruriens* var. *thekkadiensis* (1) accessions varied between 0.58 to 6.42 (%, dr. wt). *M. pruriens* var. *pruriens* elite accessions are 4283 (6.42%, wild) and 4498 (6.11%, wild) (Table 1). Other elite accessions are *M. pruriens* var. *thekkadiensis* (4147, wild, 4.34%) and *M. pruriens* var. *hirsuta* (3820, wild, 4.27%) (Table 1). Among the EP grown accessions, *M. pruriens* var. *pruriens* (4098) showed highest L-dopa content of 4.32% (Table 1). These elite accessions could be selected as candidates for multiplication and use in pharmaceutical applications. Since L-dopa is an antinutritional factor, seeds of *M. pruriens* var. *pruriens* (4072, EP grown, 0.58%; 3822, EP grown, 0.84%; 4450, wild, 0.89%; 4100, wild, 0.95%; 4090, wild, 0.97%) and *M. pruriens* var. *utilis* (4456, wild, 0.65%; 4504, wild, 0.97%; 4456, wild, 0.98%) accessions, which showed less than 1% L-dopa, can be multiplied and utilized as protein-rich diets.

We consistently found degradation patterns of L-dopa in *M. pruriens* seeds in the acidic medium of 1:1 formic acid-alcohol. On HPTLC profiling, seed extracts of all four *M. pruriens* varieties (collected from various wild locations and EP grown) showed L-dopa at R*f* 0.34 **±** 0.02 and a consistent second degradation peak at R*f* 0.41 **±** 0.02. This degraded peak was indentified as a labile mix of dopamine, dopachrome, leucodopachrome, dopaquinone and other ROS by HPTLC, DART-MS and LC/EI-MS. On an average, the degradation of L-dopa in a 24 h extraction protocol was a high 52.11%, which is significant enough to cause adverse effects in biological systems.

HPTLC profiles of *M. pruriens* var. *pruriens* seed extract (4450) and L-dopa standard in acidic, neutral and water media showed time dependent degradation. L-dopa degradation rates in the acidic medium (1:1 formic acid-alcohol) were equivalent or even higher compared to Tris buffer (neutral) or water under identical extraction periods. In seven days, *M. pruriens* var. *pruriens* seed extract and L-dopa in these liquid media resulted in degradation with gradual appearance of a black deposit. In 30 days, both *M. pruriens* var. *pruriens* seed extract and L-dopa in these solvent media resulted in significant degradation and strong black deposits. The degradation rates of L-dopa in *M. pruriens* var. *pruriens* extracts and standard L-dopa were low at 4 °C compared to room temperature.

Recent studies showed that L-dopa degradation products and dopamine adducts result in oxidative stress and cause selective cytotoxicity of neuronal cells inducing pathogenesis in PD^18^,^20^,^21^,^22^,^23^,^24^,^25^,^43^,^44^. Moreover, in PD treatment, *M. pruriens* seeds or L-dopa are administered for very long periods. Chronic L-dopa therapy in PD results in movement disorders or dyskinesia in most patients. *M. pruriens* seeds and its preparations are also used for the treatment of PD in the traditional medicinal system of Ayurveda since ancient times. Certain studies claimed that *M. pruriens* seeds are even more effective than L-dopa in PD treatment. Some literature reports caution insufficient evidence to recommend the clinical use of *M. pruriens* in the treatment of PD^45^,^46^. *M. pruriens* seeds are also used in tonics for male vitality and virility in Ayurveda^28^,^47^.

Since *M. pruriens* seeds and/or L-dopa are used for treatment of PD and as an aphrodisiac both in modern and/or traditional systems of medicine, the finding of high level of L-dopa degradation (in its pure form and in *M. pruriens* extracts) into damaging quinones and ROS is very significant. Our finding of consistent degradation products suggests the need for careful review of the processing of *M. pruriens* seeds (drying/roasting, powdering, extraction), L-dopa and their mode(s) of administration (medium, pH, temperature). Further studies are required to confirm the adverse effects of the degradation products from the ‘cure’ (*M. pruriens*, L-dopa) itself in PD patients and other users.

## Methods

### Plant materials

Seeds of thirty accessions of *M. pruriens* varieties viz. *M. pruriens* var. *pruriens* (21), *M. pruriens* var. *hirsuta* (3), *M. pruriens* var. *utilis* (5) and *M. pruriens* var. *thekkadiensis* (1) were collected in January to April 2009 from various wild locations in Kerala in south India (Table 1). GPS coordinates and other pertinent field data of these *M. pruriens* accessions were recorded during field trips. Voucher specimens of these *M. pruriens* accessions were deposited at the Herbarium (TBGT) of Jawaharlal Nehru Tropical Botanic Garden and Research Institute (JNTBGRI). Seeds of these thirty *M. pruriens* accessions collected were dried and powdered (separately).

A second set of *M. pruriens* seeds collected were initially planted in a FGB of the species established at JNTBGRI. First generation seeds of 21 accessions of *M. pruriens* var. *pruriens* and 5 accessions of *M. pruriens* var. *utilis* were collected from the FGB and planted in the EP in Randomized Block Design with two replications of each accession and one plant in each replication. *M. pruriens* var. *hirsuta* and *M. pruriens* var. *thekkadiensis* accessions did not produce fruits in the FGB. Therefore, these two varieties were not planted in the EP. *M. pruriens* var. *pruriens* and *M. pruriens* var. *utilis* accessions were maintained in the EP in uniform conditions. *M. pruriens* accessions planted in both FGB and EP were irrigated as and when required, and not supplemented with any external fertilizers. Second generation seeds of 21 accessions of *M. pruriens* var. *pruriens* and 5 accessions of *M. pruriens* var. *utilis* were collected in January to April 2011, dried and powdered (separately) (Table 1).

### L-dopa extraction

*M. pruriens* seed powder (2 g each) was extracted with 1:1 formic acid-alcohol (20 ml, 2 h) at room temperature and the extract was filtered. Seed powder residue was then repeatedly extracted with 1:1 formic acid-alcohol (3 × 10 ml, 2 h each), and extracts were filtered. This seed residue was again extracted with 1:1 formic acid-alcohol (10 ml, overnight). Filtrates (of five extractions) were pooled, centrifuged (5000 rpm, 30 min, 10 °C) and made up to 100 ml using 1:1 formic acid-alcohol. This *M. pruriens* seed extract (5 ml) was concentrated on a rotary evaporator, and the extract weight was recorded. This (concentrated) *M. pruriens* seed extract was dissolved in 20 ml 1:1 formic acid-alcohol and used for L-dopa quantification by HPTLC-densitometry. This extraction protocol was followed for quantification of L-dopa in seeds of all (56) *M. pruriens* accessions (Table 1). Extraction protocol was optimized for 24 h and cold extraction was preferred (against hot extraction) due to the labile nature (degradation) of L-dopa during extraction.

### Quantification of L-dopa

L-dopa content in *M. pruriens* seed extracts was quantified using an HPTLC (CAMAG, Switzerland) made up of Linomat V sample applicator, twin-trough plate development chamber, TLC Scanner 3 and WinCATS Software 4.03. *M. pruriens* seed extract (5 ml concentrated seed extract) was dissolved in 20 ml of 1:1 formic acid-alcohol (see L-dopa extraction), 4 μl of this solution was repeatedly applied to silica gel HPTLC plate (60 F254, E. Merck, Germany, 20 × 10 cm, 0.2 mm thickness) as 6 mm wide bands with Camag Linomat V sample applicator, fitted with a microsyringe, in N_2_ flow (application rate −50 nL/s, space between two bands −11.3 mm, slit dimension- 6 × 0.45 mm, scanning speed −20 mm/s). L-dopa standard was also applied along with *M. pruriens* seed extracts. HPTLC plate was developed upto 80 mm in the twin-trough glass chamber pre-saturated for 30 min with mobile phase butanol:acetic acid:water (4:1:1, v/v, 24 ml). Developed plate was scanned densitometrically at 282 nm (deuterium lamp) using TLC Scanner 3 equipped with WinCATS software. L-dopa at R*f* 0.34 ± 0.02 (n = 56) and a second degradation peak (SDP) at R*f* 0.41 ± 0.02 (n = 56) were found in *M. pruriens* seed extract in butanol:acetic acid:water (4:1:1, v/v) (Fig. 2a–d). Similar quantification protocol was followed for all *M. pruriens* seed extracts. Freshly dissolved L-dopa did not show the second signal, but after 24 h in solvent (1:1 formic acid-alcohol) it showed a clear second signal at R*f* 0.41 (Fig. 2a,b). Other signals in *M. pruriens* seed extracts were well resolved from these two L-dopa based signals (Fig. 2c,d). Solvent systems such as 7:3 ethanol:water, 4:1:1 butanol:acetic acid:water, 4:1:5 butanol:acetic acid:water and 4:2:1 butanol:acetic acid:water were tried for development of plates. Of these, 4:1:1 butanol:acetic acid:water gave best resolution of signals on development.

### Data analysis, validation

HPTLC-based quantification of L-dopa was validated in terms of precision, accuracy, repeatability and linearity. Specificity of the assays was tested by repeated application of standard L-dopa. R*f* values (R*f* 0.34 **±** 0.02, n = 56) of the standard was reproducible, and was found to be same as the values observed for the peak (L-dopa) in *M. pruriens* seed extracts. Calibration curve was plotted between amount of standard L-dopa (fresh) *versus* average response (peak area) (y = 6.542x + 88.22, R^2^ = 0.996). Linearity of the calibration curve in the range 100-1000 ng was ensured. Percentage L-dopa content(s) (R*f* 0.34 **±** 0.02, % **±** SD, n = 6, based on dry weight) in *M. pruriens* extracts were calculated from peak areas using the standard curve. Percentage of second degradation peak (SDP, R*f* 0.41 ± 0.02) which is a combination of labile molecules was also quantified based on L-dopa standard curve. Repeatability of sample application (instrumental precision) was assessed by applying a sample solution (*M. pruriens* extract, 4 μl) on a HPTLC plate developed up to 80 mm under saturation conditions with butanol:acetic acid:water (4:1:1, v/v) as the mobile phase in the twin-trough glass chamber (previously saturated with the solvent for 30 min). The spot (L-dopa) was scanned six times, % coefficient of variation was acceptable. Robustness of the method was checked by slightly altering the mobile phase composition and plate developing distance was checked. No considerable effect on the data was found. Recovery studies were carried out (in two modes) by the addition of L-dopa to pre-analyzed *M. pruriens* extracts and they were again analyzed (see Quantification of L-dopa). In the first mode, (i) *M. pruriens* var. *pruriens* seed powder (Acc. No. 4088, 2 g) was extracted in the five-step protocol (24 h) and (ii) standard L-dopa (10 mg) was added initially to *M. pruriens* var. *pruriens* seed powder (Acc. No. 4088, 2 g) and extracted in the five-step protocol (24 h). (i), (ii) and (iii) fresh standard L-dopa (1 μg/μl), in 1:1 formic acid-alcohol were loaded (4 μl each) onto HPTLC plate, developed with butanol:acetic acid:water (4:1:1, v/v) and peak areas were measured at 282 nm (see L-dopa extraction, Quantification of L-dopa). % recovery of L-dopa was calculated from peak areas as 49.78%. In the second mode, *M. pruriens* var. *pruriens* seed powder (Acc. No. 4502, 2 g) was extracted in the standard five-step protocol (24 h). (i) *M. pruriens* var. *pruriens* extract in 1:1 formic acid-alcohol (4 μl), (ii) *M. pruriens* var. *pruriens* extract in 1:1 formic acid-alcohol (4 μl) and fresh L-dopa dissolved in 1:1 formic acid-alcohol (1 μg/μl, 4 μl) and (iii) fresh L-dopa dissolved in 1:1 formic acid-alcohol (1 μg/μl, 4 μl) were loaded onto HPTLC plate ((ii) co-spotted), developed and peak areas were measured. % L-dopa recovery was calculated as 99.30%. % residual standard deviations (RSD) were determined as 2.63 (L-dopa) and 3.58 (SDP). Limit of detection (LOD, average of 3.3 × SD of peak area/slope of calibration curve for 56 accessions, n = 6) and limit of quantification (LOQ, average of 10 × SD of peak area/slope of calibration curve for 56 accessions, n = 6) were determined for both L-dopa (LOD −31.46 ng, LOQ −95.32 ng) and SDP (LOD −21.10 ng, LOQ −63.93 ng)^48^,^49^.

### L-dopa degradation

*M. pruriens* var. *pruriens* seed extract (Acc. No. 4450) prepared by the five stage extraction for 24 h (fresh *M. pruriens* var. *pruriens* seed extract) and standard L-dopa (fresh) were suspended in 1:1 formic acid-alcohol and kept at room temperature for seven days with occasional stirring. These resulted in decomposed *M. pruriens* var. *pruriens* seed extract and decomposed L-dopa standard, respectively. Fresh *M. pruriens* var. *pruriens* seed extract, freshly prepared L-dopa standard (both in 1:1 formic acid-alcohol), decomposed *M. pruriens* var. *pruriens* seed extract and decomposed L-dopa standard were applied onto silica gel plates (60 F254, E. Merck, Germany, 20 × 10 cm, 0.2 mm thickness) by HPTLC (CAMAG, Switzerland), developed in butanol:acetic acid:water (4:1:1, v/v, 24 ml) and scanned at 282 nm (TLC Scanner 3, CAMAG, Switzerland) (Fig. 2).

Fresh *M. pruriens* var. *pruriens* seed extract (100 mg), decomposed *M. pruriens* var. *pruriens* seed extract (100 mg) and decomposed L-dopa standard (26.1 mg) were analyzed by DART-MS on an AccuTOF JMS-T100LC Mass Spectrometer having a DART (JEOL, USA). Samples were analyzed directly in front of the DART source. Dry He was used at a flow rate of 4 LPM for ionization at 350 °C. Orifice 1 was set at 28 V, spectra were collected, and the data from 6-8 scans were averaged (Fig. S1-S3). Again, decomposed *M. pruriens* var. *pruriens* seed extract (80 mg) and decomposed L-dopa standard (20 mg) were subjected to LC/ESI-MS analysis on a Surveyor-LCQ Deca XP plus system (Thermo Finnigan, USA) with Hypersil BDS C18 column (length 250 mm, int. dia. 4.6 mm, particle size 5 μm), mobile phase: methanol-water 85:15, inj. vol.: 10 μl, flow rate: 0.3 ml/min, run time: 30 min, LC detection: PDA/UV detector, 280 nm and mass detection: electro spray ionization (Fig. S4-S7).

### Effect of pH on L-dopa degradation

*M. pruriens* var. *pruriens* (accession number 4450) seeds (1 g each) were separately extracted with 20 ml (each) of 1:1 formic acid-alcohol (strongly acidic), 20 mM Tris-HCl, 20 mM KCl (pH 7.2, neutral) and water at room temperature and at 4 °C. Similarly, standard L-dopa (5 mg) was extracted (dissolved) in 10 ml each of these three solvents at room temperature and at 4 °C. These *M. pruriens* var. *pruriens* seed/L-dopa extracts (3 μl each) were profiled using HPTLC-densitometry (as described in Quantification of L-dopa) at various time periods viz., 1 h after initiation of extraction, 1, 7 and 30 days after initiation of extraction (Fig. S8-S19).

## Additional Information

**How to cite this article**: Pulikkalpura, H. *et al*. Levodopa in *Mucuna pruriens* and its degradation. *Sci. Rep*. **5**, 11078; doi: 10.1038/srep11078 (2015).

## Supplementary Material

Supplementary Information

## Figures and Tables

**Figure 1:**
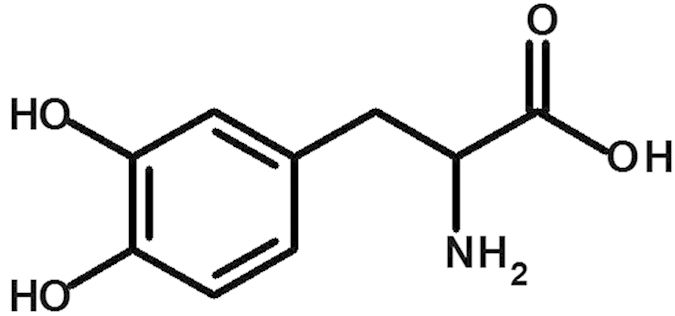
Levodopa.

**Table 1:** Quantification of L-dopa and its degradation in seeds of fifty-six accessions of four *Mucuna pruriens* varieties by HPTLC-densitometry.

Acc. No.	Collection location, district	Altitude (m)	Longitude	Latitude	Wild L-dopa (%, dr. wt.)	Wild, SDP (%, dr. wt.)^+^	EP, L-dopa (%, dr. wt.)	EP, SDP (%, dr. wt.)^+^
***Mucuna pruriens*var.*pruriens***								
3656	Wandoor, Malappuram	65	E 76° 13.662’	N 11° 11.621’	3.42 ± 0.06	1.21 ± 0.00	2.17 ± 0.0014	0.30 ± 0.00
3666	Erimayur, Palakkad	90	E 76° 34.015’	N 10° 39.409’	2.64 ± 0.00	1.27 ± 0.00	1.54 ± 0.0069	2.88 ± 0.00
3822	Achenkovil, Kollam	111	E 77° 7.267’	N 09° 05.549’	3.37 ± 0.00	1.11 ± 0.00	0.84 ± 0.0087	2.93 ± 0.0243
3911	Payannur, Kannur	63	E 75° 5.844’	N 12° 18.416’	3.49 ± 0.00	0.34 ± 0.00	1.44 ± 0.00	3.27 ± 0.00
3912	Kappad, Kozhikode	1	E 75° 42.833’	N 11° 23.599’	1.63 ± 0.00	1.15 ± 0.00	1.04 ± 0.00	3.34 ± 0.00
4072	Mathur, Palakkad	125	E 76° 33.646’	N 10° 44.656’	3.51 ± 0.00	2.05 ± 0.00	0.58 ± 0.00	1.77 ± 0.0134
4077	Panathur, Kasaragod	123	E 75° 22.719’	N 12° 29.267’	2.34 ± 0.00	0.41 ± 0.00	2.54 ± 0.00	0.67 ± 0.00
4088	Malampuzha, Palakkad	135	E 76° 43.374’	N 10° 49.401’	3.03 ± 0.0014	0.15 ± 0.00	3.30 ± 0.0071	0.36 ± 0.00
4090	Elavenchery, Palakkad	93	E 76° 39.091’	N 10° 35.758’	0.97 ± 0.00	2.75 ± 0.00	2.93 ± 0.00	0.45 ± 0.00
4091	Pulpally, Wayanad	802	E 76° 09.662’	N 11° 47.655’	1.66 ± 0.00	2.96 ± 0.00	2.12 ± 0.0027	0.00 ± 0.00
4098	Pala, Kottayam	240	E 76° 38.660’	N 09° 44.285’	2.60 ± 0.00	0.79 ± 0.00	4.32 ± 0.0497	0.00 ± 0.00
4100	Muthalamada, Palakkad	143	E 76° 45.530’	N 10° 36.595’	0.95 ± 0.00	3.19 ± 0.00	2.83 ± 0.0035	0.65 ± 0.00
4101	Madavoor, Kozhikode	60	E 75° 44.110’	N 11° 21.523’	3.03 ± 0.00	0.58 ± 0.00	1.22 ± 0.00	3.16 ± 0.00
4283	Govindapuram, Palakkad	157	E 076° 49.002’	N 10° 36.866’	6.42 ± 0.0034	0.00 ± 0.00	1.21 ± 0.0024	2.16 ± 0.0320
4290	Changanassery, Kottayam	90	E 076° 32.247’	N 09° 26.386’	2.61 ± 0.00	0.49 ± 0.00	3.99 ± 0.00	0.11 ± 0.00
4292	Mala, Thrissur	46	E 076° 16.015’	N 10° 14.592’	2.86 ± 0.00	0.49 ± 0.00	3.32 ± 0.00	0.45 ± 0.00
4448	Thamarassery, Kozhikode	14	E 075° 54.801’	N 11° 23.099’	3.01 ± 0.00	1.60 ± 0.00	1.14 ± 0.00	2.73 ± 0.00
4449	Umathoor, Malappuram	56	E 076°04.949’	N 11° 01.920’	2.27 ± 0.00	0.71 ± 0.00	2.89 ± 0.00	0.58 ± 0.00
4450	Koothattukulam, Ernakulam	52	E 076°35.763’	N 09° 52.641’	0.89 ± 0.00	2.45 ± 0.00	1.04 ± 0.00	2.93 ± 0.00
4498	Karyavattom, Thiruvananthapuram	15	E 076° 52.386’	N 08° 33.038’	6.11 ± 0.00	0.10 ± 0.00	3.05 ± 0.0065	0.64 ± 0.0059
4503	Alappuzha, Alappuzha	10	E 076°19.185’	N 09° 29.106’	1.78 ± 0.00	3.85 ± 0.00	2.50 ± 0.00	1.15 ± 0.00
***Mucuna pruriens*var.*hirsuta***								
3820	Pullupara, Idukki	755	E 76° 58.242’	N 09° 33.280’	4.27 ± 0.00	0.01 ± 0.00	*	*
4099	Silent Valley, Palakkad	827	E 76° 30.764’	N 11° 04.431’	1.01 ± 0.00	1.14 ± 0.00	*	*
4282	Ponmudi, Thiruvananthapuram	256	E 77° 05.504’	N 08° 43.613’	3.29 ± 0.00	1.40 ± 0.00	*	*
***Mucuna pruriens*var.*utilis***								
4446	Thamarassery, Kozhikode	66	E 075°54.967’	N 11° 22.835’	2.67 ± 0.0033	0.35 ± 0.00	3.70 ± 0.00	0.00 ± 0.00
4456	Malampuzha, Palakkad	120	E 076°39.261’	N 10° 52.333’	0.98 ± 0.00	0.12 ± 0.00	3.97 ± 0.00	0.14 ± 0.00
4502	Ambalapuzha, Alappuzha	68	E 076°25.139’	N 09° 20.235’	0.65 ± 0.00	2.88 ± 0.00	1.33 ± 0.00	0.77 ± 0.00
4504	Cherthala, Alappuzha	65	E 076 ° 19.487’	N 09 ° 41.423’	0.97 ± 0.00	3.82 ± 0.00	2.22 ± 0.00	0.00 ± 0.00
4505	Cherthala, Alappuzha	65	E 076 ° 19.487’	N 09 ° 41.423’	1.09 ± 0.00	3.26 ± 0.00	3.36 ± 0.00	0.07 ± 0.00
***Mucuna pruriens*var.** ***thekkadiensis***								
4147	Thekkady, Idukky	860	E 77° 10.027’	N 09° 35.572’	4.34 ± 0.00	0.01 ± 0.00	*	*

SDP - Second Degradation Peak; ^+^SDP % calculated based on L-dopa standard curve; EP - Experimental Plot; ^*^Fruiting absent in FGB grown accessions. Each percentage value is an average of six values.
