# Supplementary material for: Levodopa in Mucuna pruriens and its degradation
Source: Sci Rep. 2015 Jun 9;5:11078. doi: 10.1038/srep11078 (PMC4460905; doi:10.1038/srep11078)
Supplement: Supplementary Information [file srep11078-s1.doc]

**SUPPLEMENTARY INFORMATION**

**Levodopa in *Mucuna pruriens* and its degradation**

**Haridas Pulikkalpura^1^, Rajani Kurup^2^, Paravanparampil Jacob Mathew^1^, Sabulal Baby^2^***

^1^Plant Genetic Resource Division, Jawaharlal Nehru Tropical Botanic Garden and Research Institute, Pacha-Palode, Thiruvananthapuram 695 562, Kerala, India, ^2^Phytochemistry and Phytopharmacology Division, Jawaharlal Nehru Tropical Botanic Garden and Research Institute, Pacha-Palode, Thiruvananthapuram 695 562, Kerala, India

Correspondence and requests for materials should be addressed to S.B. (sabulal@gmail.com)

***Mucuna pruriens* is the best known natural source of L-dopa, the gold standard for treatment of Parkinsonism. *M. pruriens* varieties are protein rich supplements, and are used as food and fodder worldwide. Here, we report L-dopa contents in seeds of fifty six accessions of four *M. pruriens* varieties, *M. pruriens* var. *pruriens*, *M. pruriens* var. *hirsuta*, *M. pruriens* var. *utilis* and *M. pruriens* var. *thekkadiensis*, quantified by HPTLC-densitometry. L-dopa contents varied between 0.58 to 6.42 (%, dr. wt.). High and low L-dopa yielding genotypes/chemotypes of *M. pruriens* could be multiplied for medicinal and nutritional purposes, respectively. HPTLC profiles of *M. pruriens* seeds on repeated extraction (24 h) in 1:1 formic acid-alcohol followed by development in butanol:acetic acid:water (4:1:1, v/v) showed consistent degradation of L-dopa (R*f* 0.34 ± 0.02) into a second peak (R*f* 0.41 ± 0.02). An average of 52.11% degradation of L-dopa was found in seeds of *M. pruriens* varieties. Since *M. pruriens* seeds and/or L-dopa are used for treatment of Parkinson’s disease and as an aphrodisiac both in modern and/or traditional systems of medicine, the finding of high level of L-dopa degradation (in pure form and in *M. pruriens* extracts) into damaging quinones and ROS is very significant.**


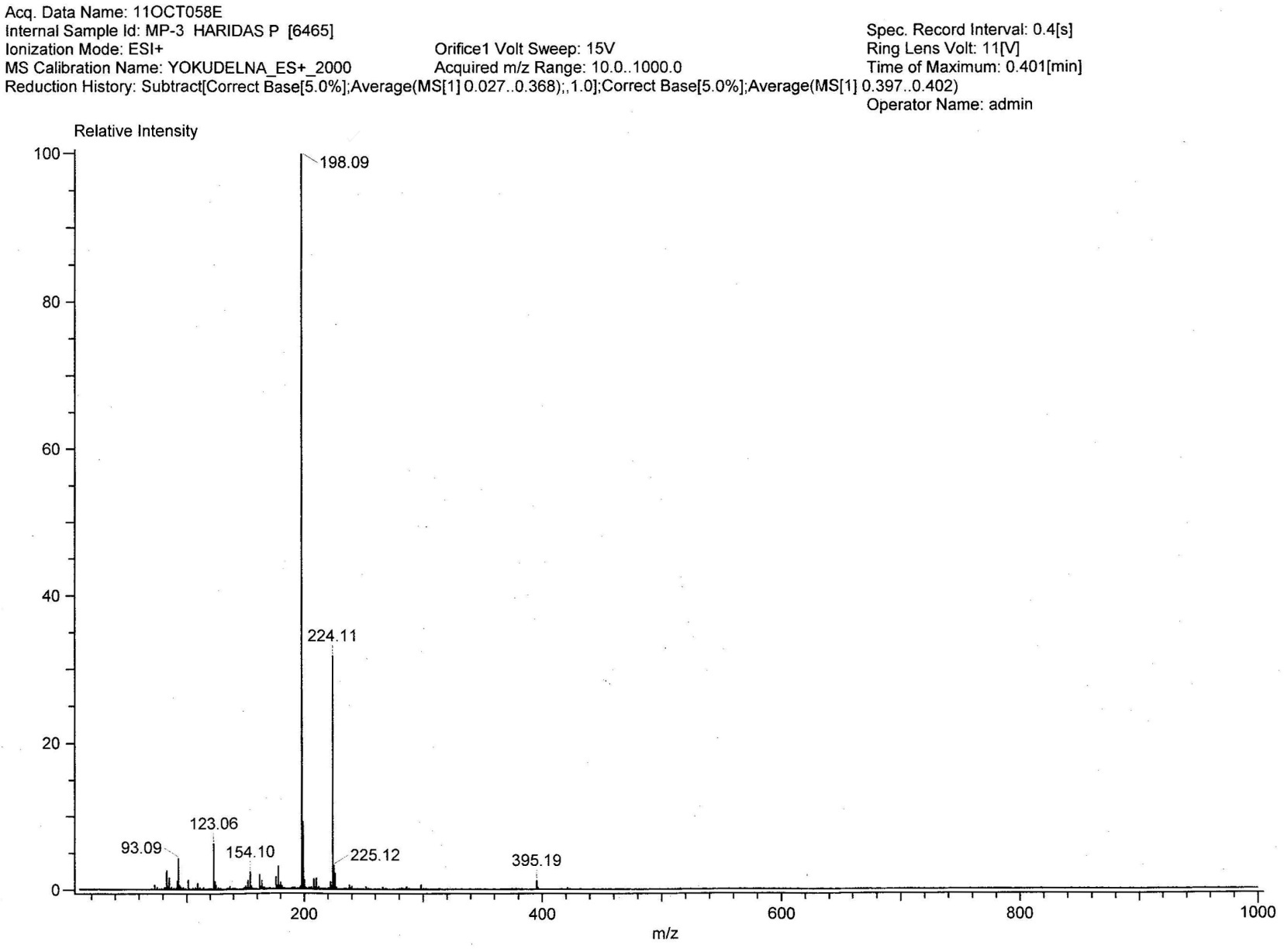


**Fig. S1.** DART-MS of fresh L-dopa.


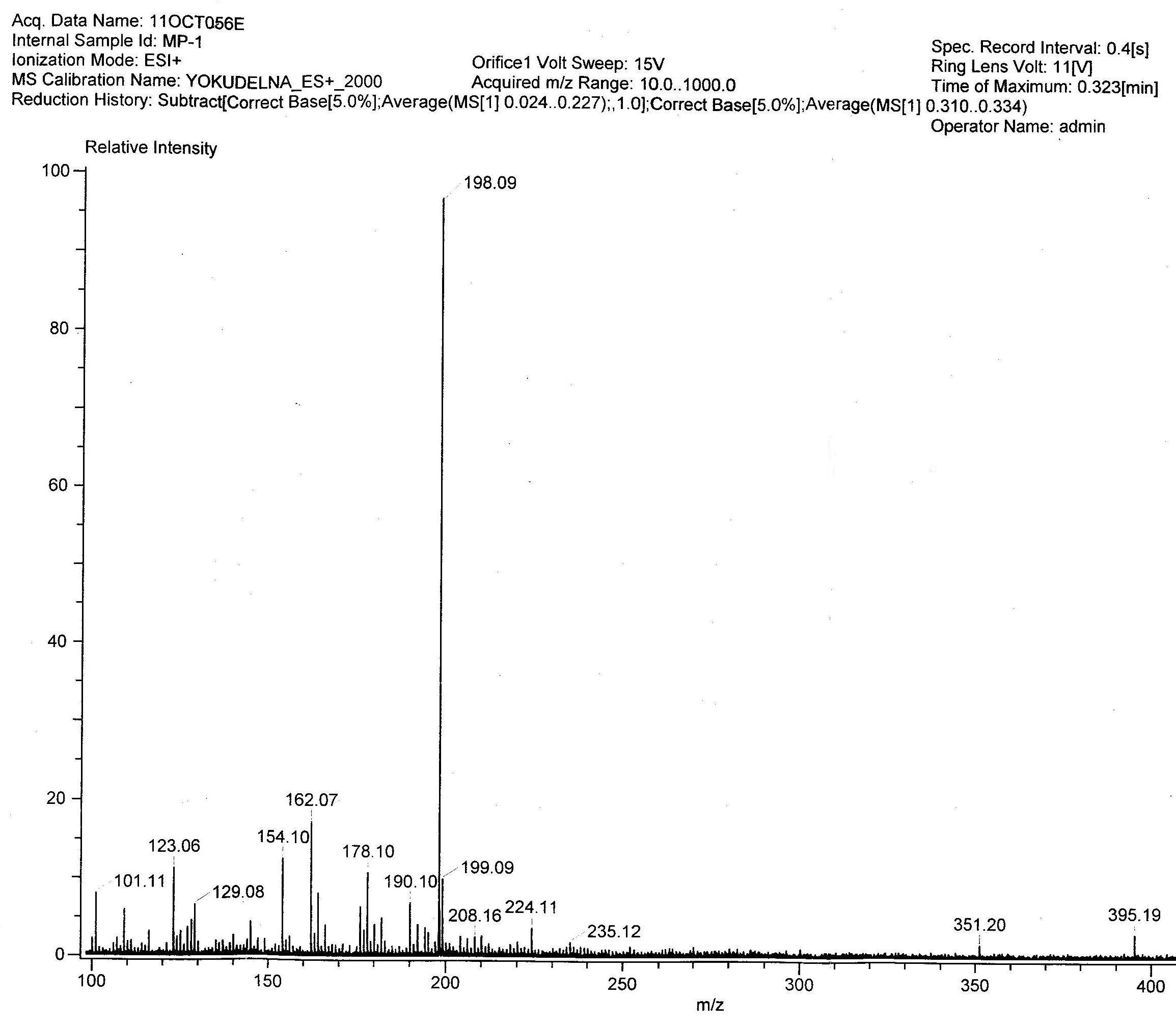


**Fig. S2.** DART-MS of fresh *Mucuna pruriens* var. *pruriens* seed extract (accession number 4450).


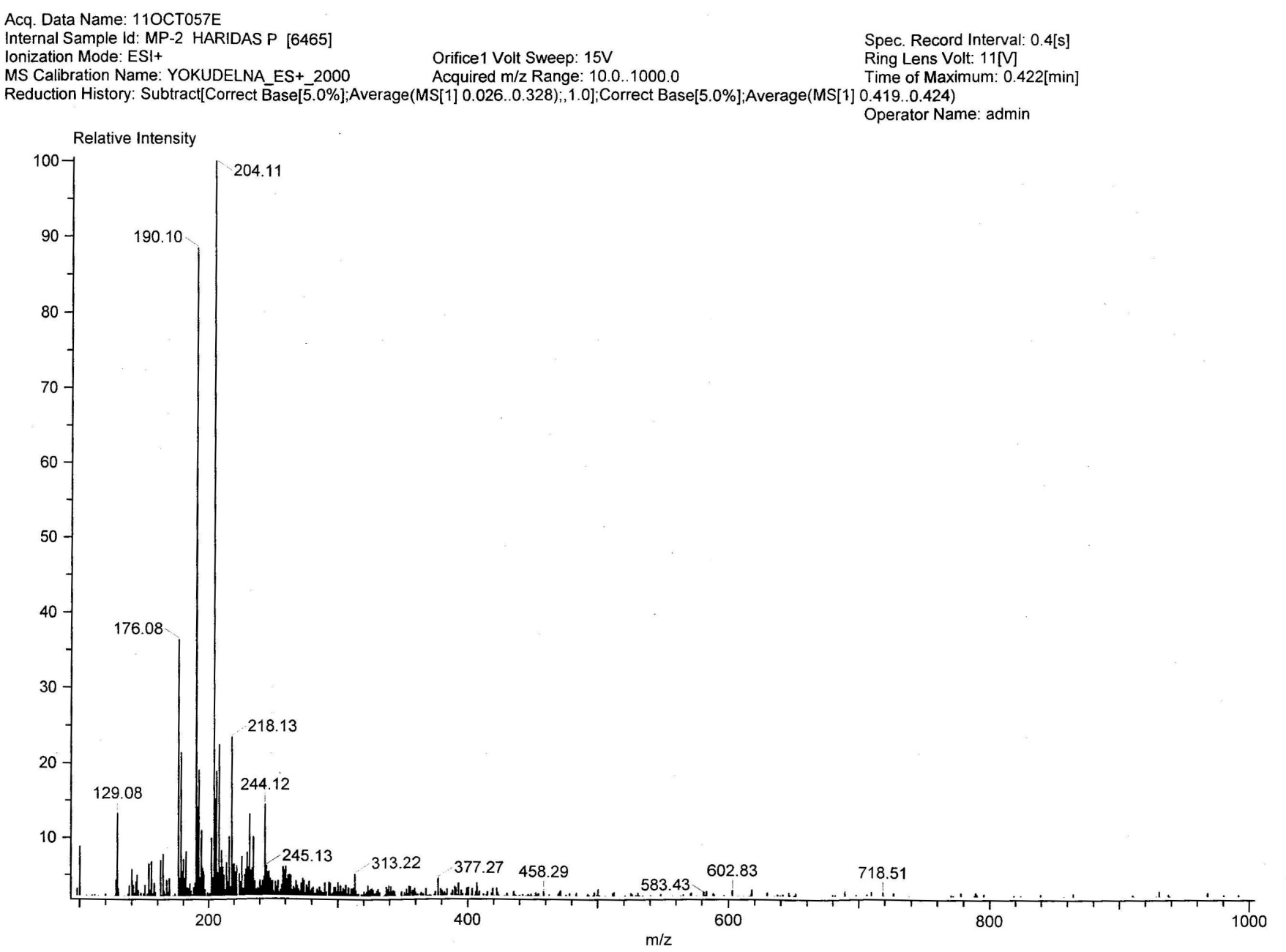


**Fig. S3.** DART-MS of decomposed *Mucuna pruriens* var. *pruriens* seed extract (accession number 4450).

**Fig. S4.** LC/EI-MS of decomposed L-dopa standard (1).

**Fig. S5.** LC/EI-MS of decomposed L-dopa standard (2).

**Fig. S6.** LC/EI-MS of decomposed L-dopa standard (3).

**Fig. S7.** LC/EI-MS of decomposed extract of *Mucuna pruriens* var. *pruriens* seed extract (accession number 4450).


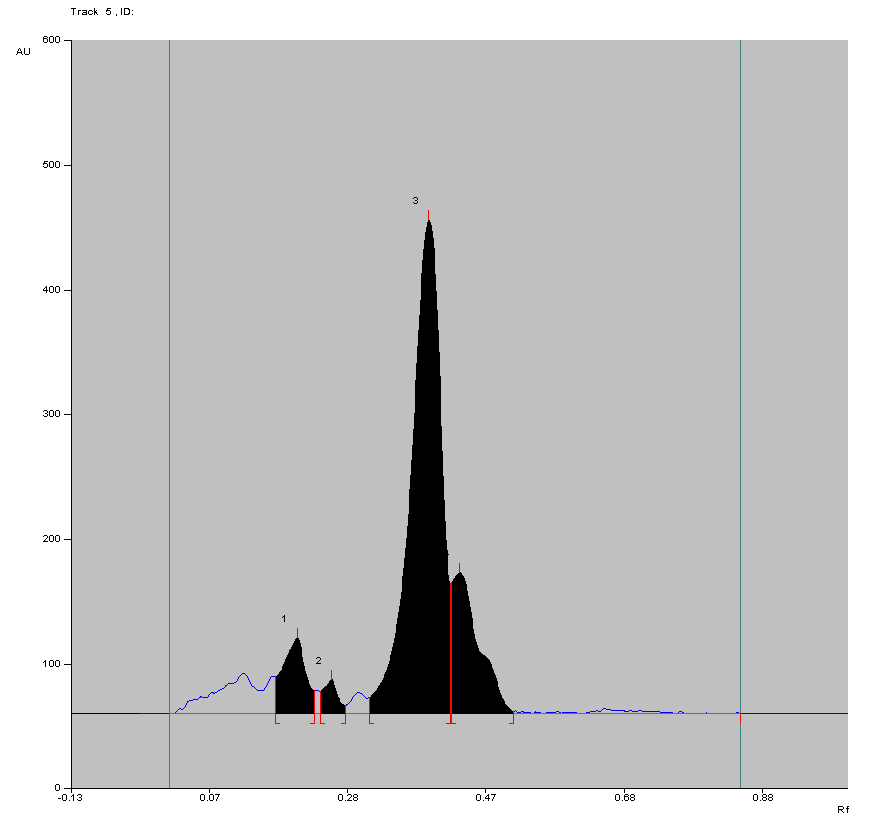


**Fig. S8.** HPTLC profile of *Mucuna pruriens* var. *pruriens* seed extract (accession number 4450) 1 h after initiation of extraction in 1:1 formic acid:alcohol.


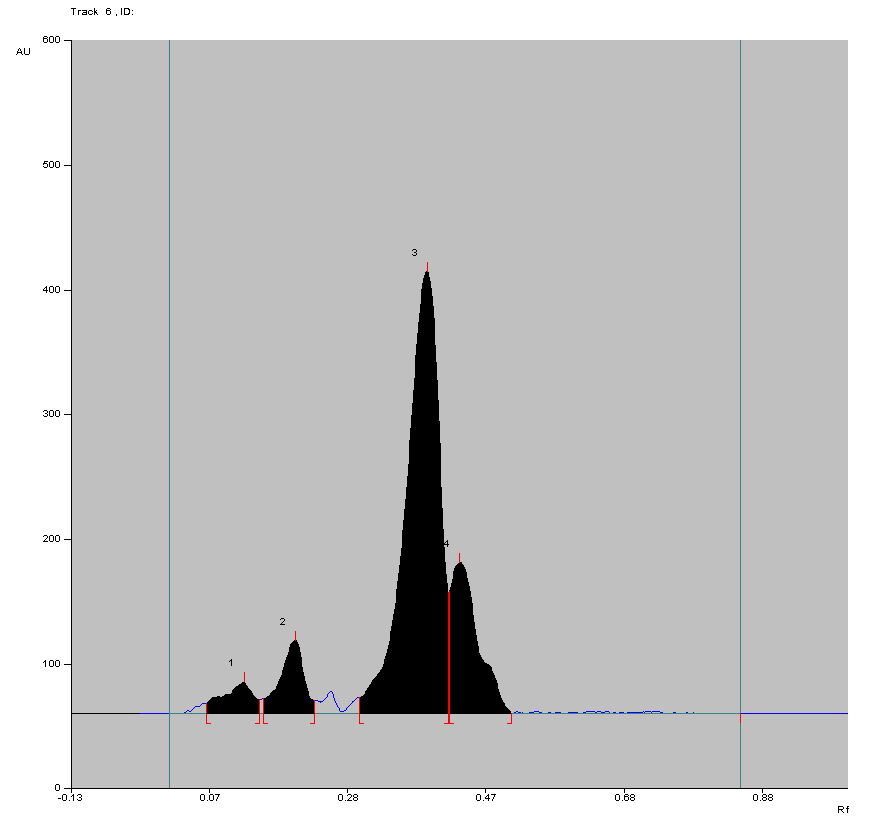


**Fig. S9.** HPTLC profile of *Mucuna pruriens* var. *pruriens* seed extract (accession number 4450) 1 h after initiation of extraction in 20 mM Tris buffer (pH 7.2).


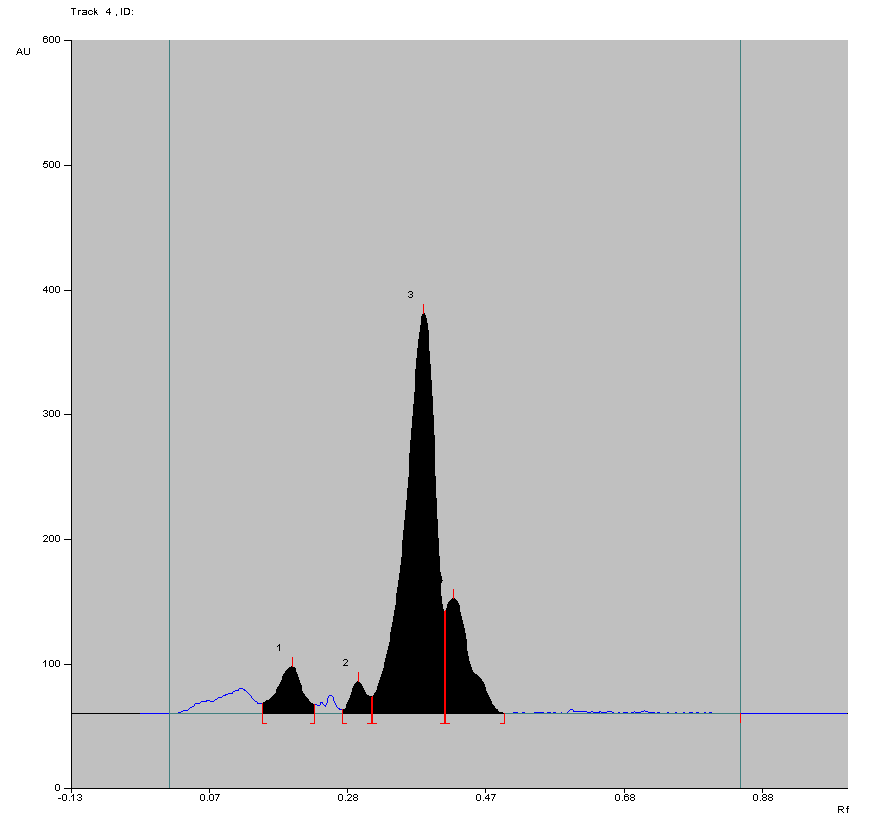


**Fig. S10.** HPTLC profile of *Mucuna pruriens* var. *pruriens* seed extract (accession number 4450) 1 h after initiation of extraction in water.


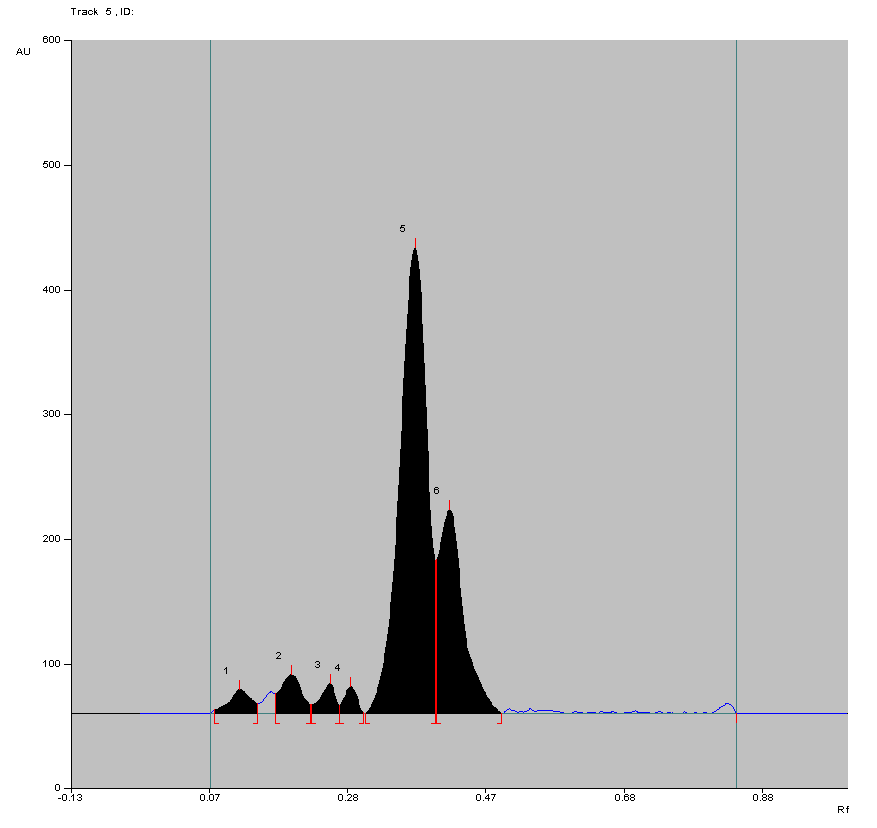


**Fig. S11.** HPTLC profile of *Mucuna pruriens* var. *pruriens* seed extract (accession number 4450) 1 day after initiation of extraction in 1:1 formic acid:alcohol.


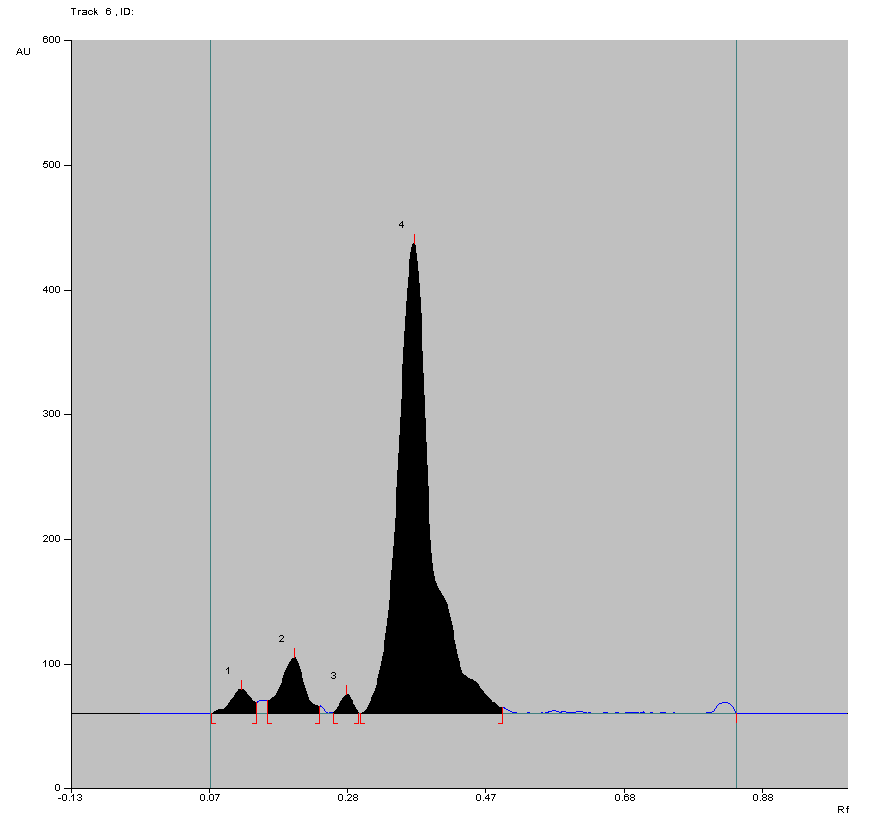


**Fig. S12.** HPTLC profile of *Mucuna pruriens* var. *pruriens* seed extract (accession number 4450) 1 day after initiation of extraction in 20 mM Tris buffer (pH 7.2).


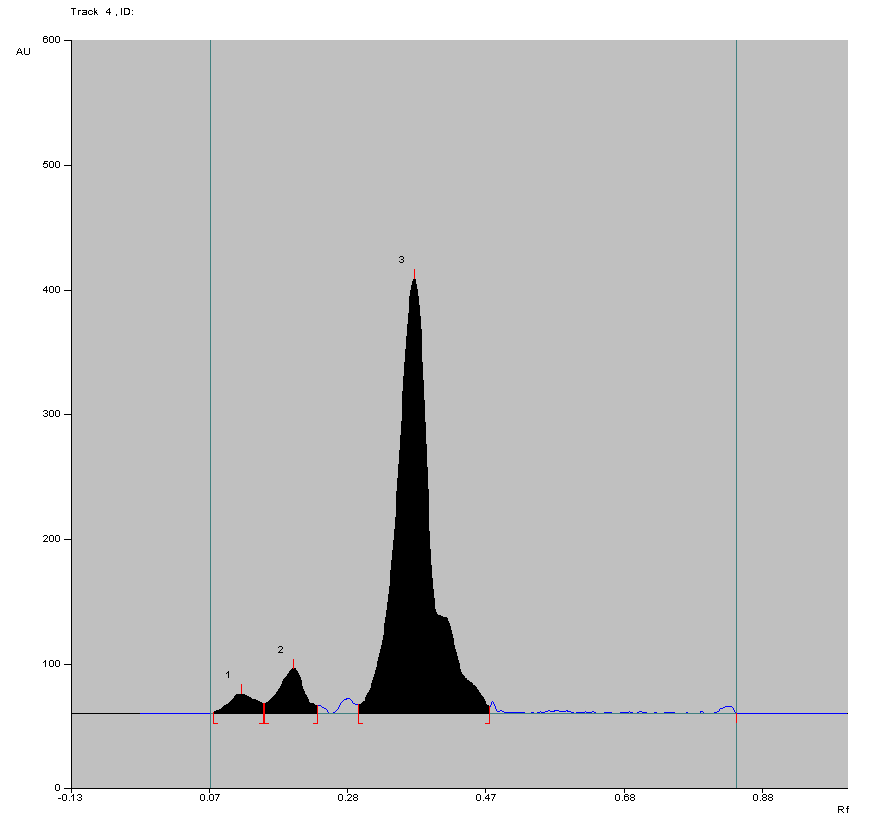


**Fig. S13.** HPTLC profile of *Mucuna pruriens* var. *pruriens* seed extract (accession number 4450) 1 day after initiation of extraction in water.


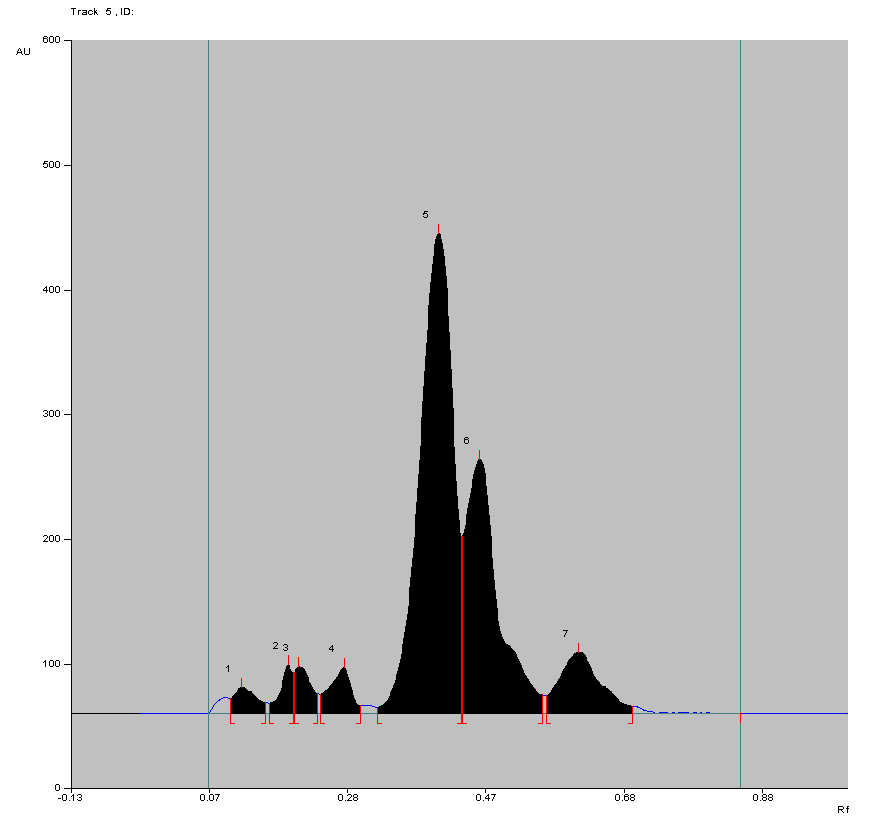


**Fig. S14.** HPTLC profile of *Mucuna pruriens* var. *pruriens* seed extract (accession number 4450) 7 days after initiation of extraction in 1:1 formic acid:alcohol.


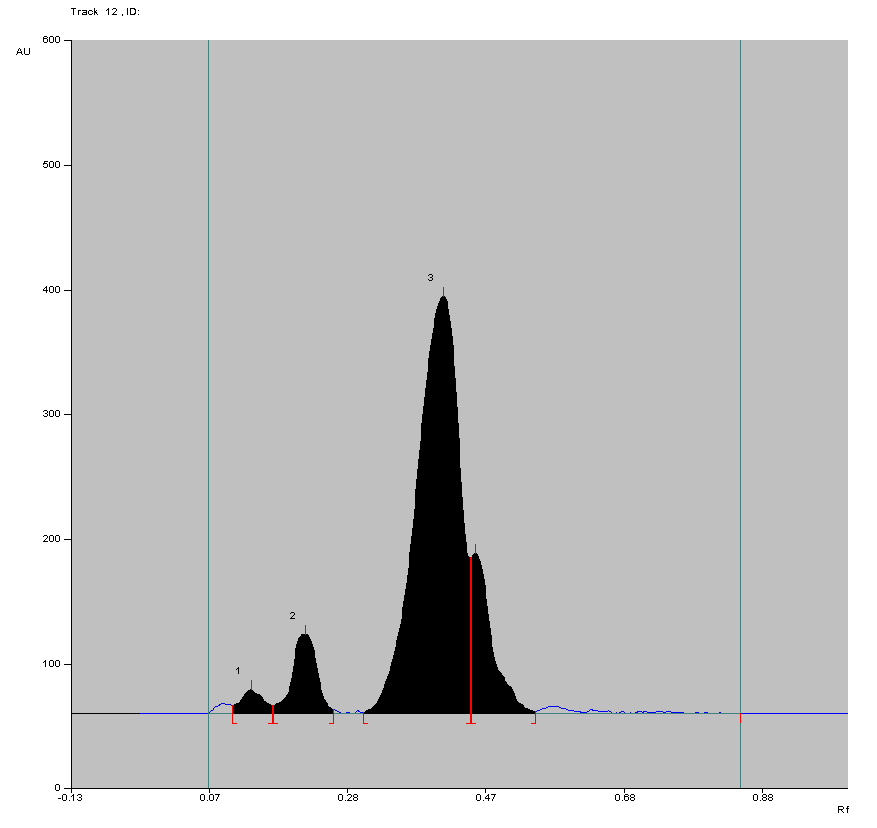


**Fig. S15.** HPTLC profile of *Mucuna pruriens* var. *pruriens* seed extract (accession number 4450) 7 days after initiation of extraction in 20 mM Tris buffer (pH 7.2).


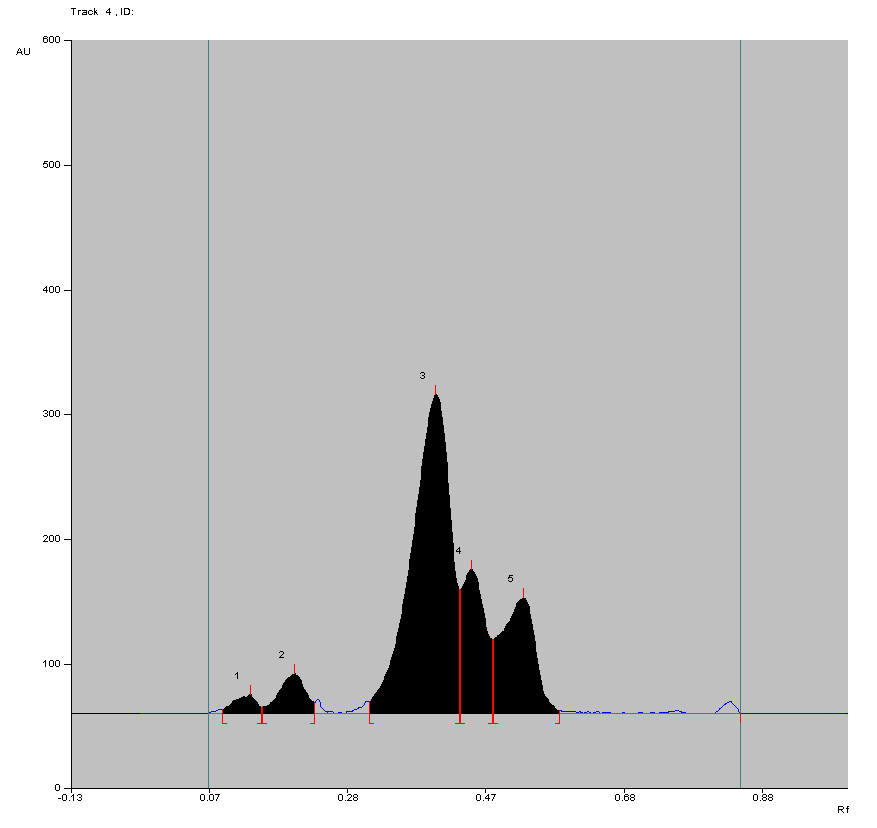


**Fig. S16.** HPTLC profile of *Mucuna pruriens* var. *pruriens* seed extract (accession number 4450) 7 days after initiation of extraction in water.


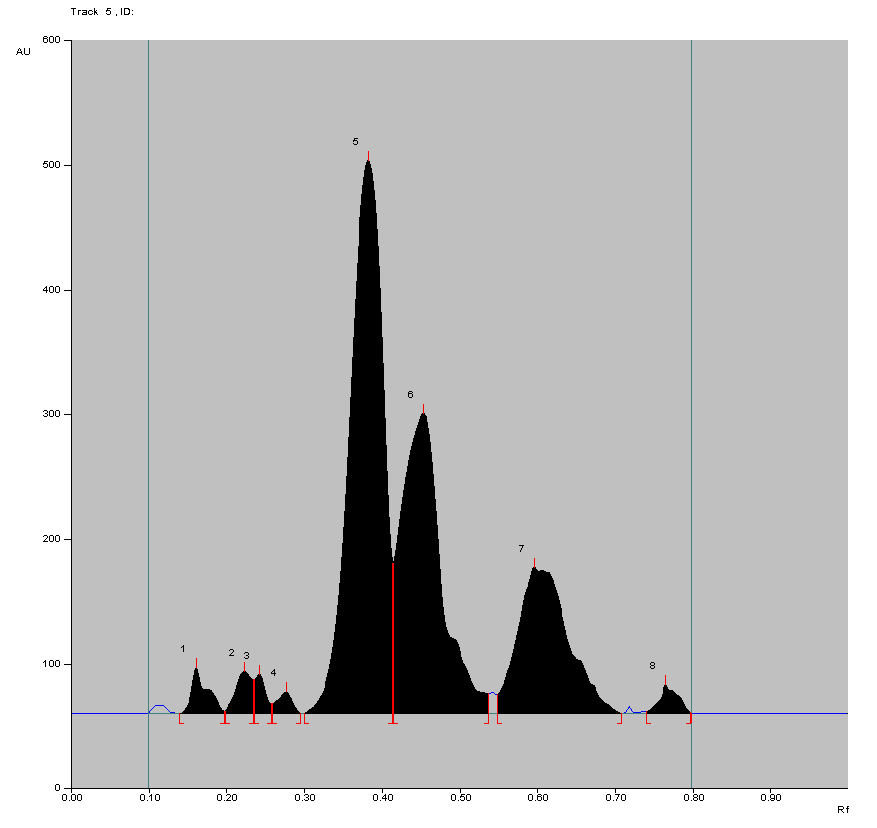


**Fig. S17.** HPTLC profile of *Mucuna pruriens* var. *pruriens* seed extract (accession number 4450) 30 days after initiation of extraction in 1:1 formic acid:alcohol.


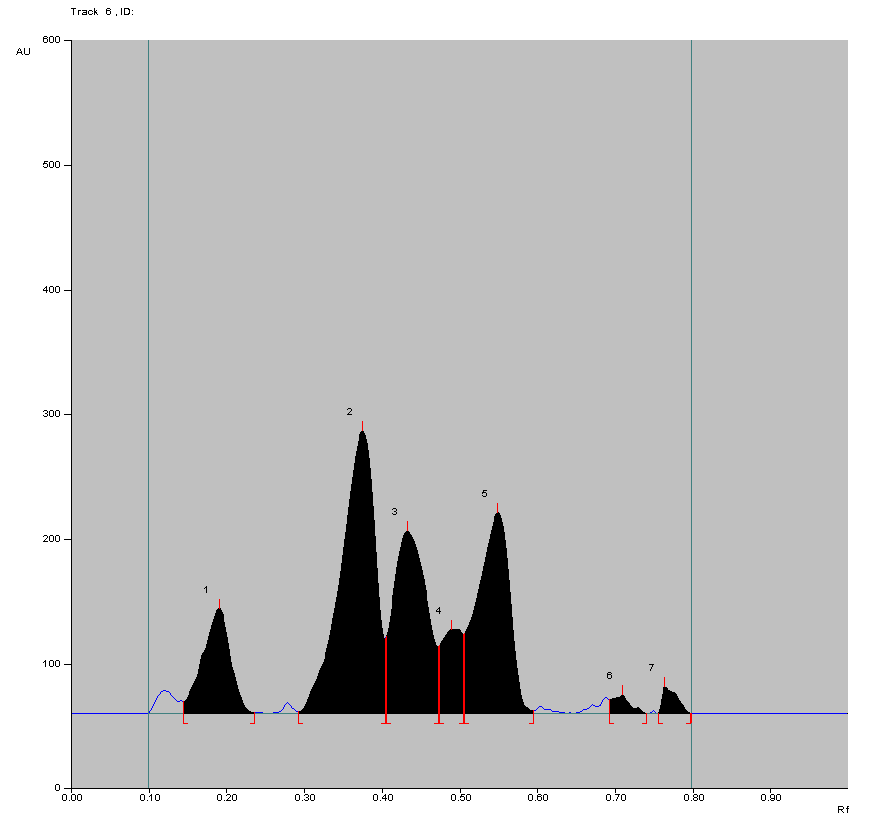


**Fig. S18.** HPTLC profile of *Mucuna pruriens* var. *pruriens* seed extract (accession number 4450) 30 days after initiation of extraction in 20 mM Tris buffer (pH 7.2).


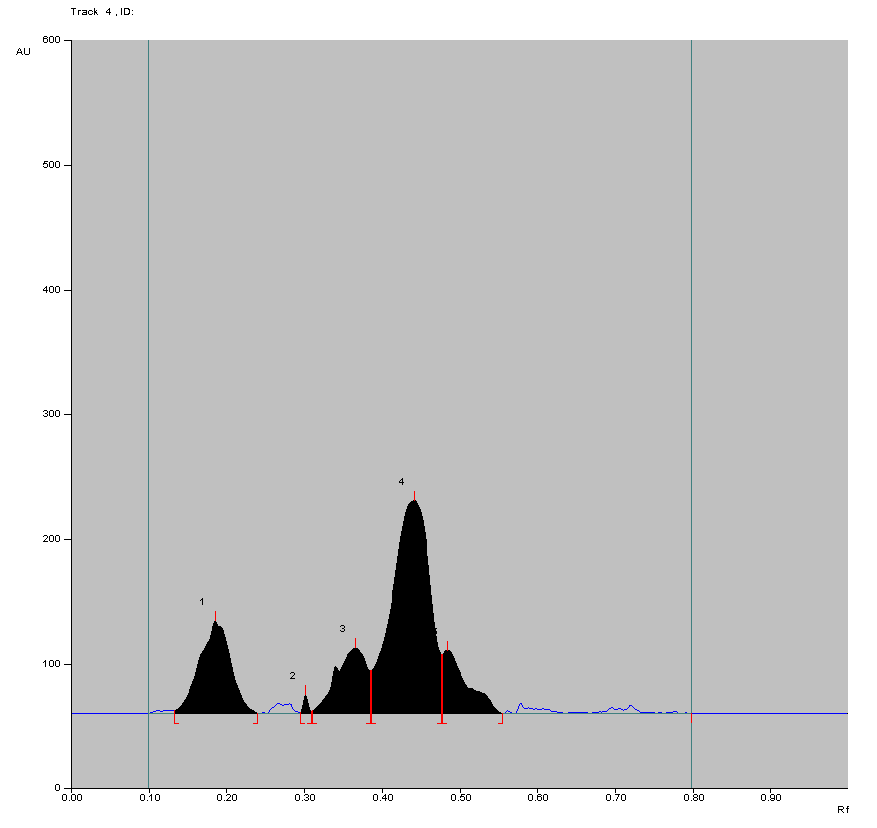


**Fig. S19.** HPTLC profile of *Mucuna pruriens* var. *pruriens* seed extract (accession number 4450) 30 days after initiation of extraction in water.
